# Saliva RNA biomarkers predict concussion duration and detect symptom recovery: a comparison with balance and cognitive testing

**DOI:** 10.1007/s00415-021-10566-x

**Published:** 2021-05-24

**Authors:** Gregory Fedorchak, Aakanksha Rangnekar, Cayce Onks, Andrea C. Loeffert, Jayson Loeffert, Robert P. Olympia, Samantha DeVita, John Leddy, Mohammad N. Haider, Aaron Roberts, Jessica Rieger, Thomas Uhlig, Chuck Monteith, Frank Middleton, Scott L. Zuckerman, Timothy Lee, Keith Owen Yeates, Rebekah Mannix, Steven Hicks

**Affiliations:** 1 Department of Pediatrics, Penn State College of Medicine, Hershey, PA 17033 USA; 2 Department of Family Medicine, Penn State College of Medicine, Hershey, PA 17033 USA; 3 Department of Emergency Medicine, Penn State College of Medicine, Hershey, PA 17033 USA; 4 Quadrant Biosciences, Syracuse, NY 13210 USA; 5 SUNY Buffalo Jacobs School of Medicine and Biomedical Sciences, UBMD Orthopaedics and Sports Medicine, Buffalo, NY 14214 USA; 6 Vanderbilt Sports Concussion Center, Vanderbilt University Medical Center, Nashville, TN 37212 USA; 7 Colgate University, Hamilton, NY 13346 USA; 8 Division of Emergency Medicine, Boston Children’s Hospital, Harvard Medical School, Boston, MA USA; 9 Department of Neuroscience and Physiology, SUNY Upstate Medical University, Syracuse, NY 13210 USA; 10 Department of Adena Regional Medical Center, Adena Bone and Joint Center, Chillicothe, OH 45601 USA; 11 Department of Psychology, Alberta Children’s Hospital Research Institute, Hotchkiss Brain Institute, University of Calgary, Calgary, AB T2N 1N4 Canada

**Keywords:** traumatic brain injury, microRNA, spit, return to play, prognosis, mTBI

## Abstract

**Objective:**

The goals of this study were to assess the ability of salivary non-coding RNA (ncRNA) levels to predict post-concussion symptoms lasting ≥ 21 days, and to examine the ability of ncRNAs to identify recovery compared to cognition and balance.

**Methods:**

RNA sequencing was performed on 505 saliva samples obtained longitudinally from 112 individuals (8–24-years-old) with mild traumatic brain injury (mTBI). Initial samples were obtained ≤ 14 days post-injury, and follow-up samples were obtained ≥ 21 days post-injury. Computerized balance and cognitive test performance were assessed at initial and follow-up time-points. Machine learning was used to define: (1) a model employing initial ncRNA levels to predict persistent post-concussion symptoms (PPCS) ≥ 21 days post-injury; and (2) a model employing follow-up ncRNA levels to identify symptom recovery. Performance of the models was compared against a validated clinical prediction rule, and balance/cognitive test performance, respectively.

**Results:**

An algorithm using age and 16 ncRNAs predicted PPCS with greater accuracy than the validated clinical tool and demonstrated additive combined utility (area under the curve (AUC) 0.86; 95% CI 0.84–0.88). Initial balance and cognitive test performance did not differ between PPCS and non-PPCS groups (*p* > 0.05). Follow-up balance and cognitive test performance identified symptom recovery with similar accuracy to a model using 11 ncRNAs and age. A combined model (ncRNAs, balance, cognition) most accurately identified recovery (AUC 0.86; 95% CI 0.83–0.89).

**Conclusions:**

ncRNA biomarkers show promise for tracking recovery from mTBI, and for predicting who will have prolonged symptoms. They could provide accurate expectations for recovery, stratify need for intervention, and guide safe return-to-activities.

**Supplementary Information:**

The online version contains supplementary material available at 10.1007/s00415-021-10566-x.

## Introduction

Guidelines from the Centers for Disease Control and Prevention (CDC) Pediatric Mild Traumatic Brain Injury (mTBI) Workgroup recommend that clinicians inform patients and families that some factors predict risk for persistent post-concussion symptoms (PPCS), but individual recovery from mTBI is unique [1]. Providers should use a combination of tools when assessing mTBI recovery (i.e., symptom scales, cognitive and balance tests). However, the workgroup also recognized these tools are insufficient to accurately predict recovery, stating, “No factors can individually predict recovery of symptoms and outcome…much of the variance in outcome remains unaccounted for, even when multiple factors are considered” [1].

The best available tool predicts PPCS with an area under the curve (AUC) of 0.68 [32]. More complicated tools to identify PPCS may require time and expertise that may preclude their use by the majority of health care providers [2, 3]. In ambulatory clinics, where patient visits may last only 15 min, rapid, objective measures that do not require specialist interpretation are urgently needed. Such tools would improve care for patients with PPCS in two ways: (1) individuals who receive education about prognosis have improved outcomes [4, 5]; and (2) identifying those at risk for PPCS provides an opportunity for early intervention prior to the development of prolonged and debilitating symptoms [1].

Non-coding ribonucleic acids (ncRNAs) represent a potential biomarker for PPCS. Several classes of ncRNAs have been implicated in concussion [6], but the best-studied are microRNAs (miRNAs) [7]. miRNAs are 19–23 base-pair nucleic acid fragments that block translation of specific proteins in response to environmental changes, such as a concussion [8]. Studies of animal models [9, 10] and human adults [6, 11] have reported changes in serum and saliva miRNA expression following traumatic brain injury (TBI). Salivary changes in miRNAs mirror cerebrospinal fluid miRNA patterns, and may aid identification of TBI [12, 13]. Moreover, peripheral miRNA alterations persist over time [9]. Our pilot investigation, involving 52 youth with mTBI, demonstrated that five miRNAs in saliva could be used to accurately predict PPCS [14]. However, the longitudinal relationship between saliva miRNAs and functional measures of balance and cognition has not been assessed in relationship to symptom duration and recovery.

The goals of the study were to: (1) determine the ability of salivary ncRNAs measured within 14 days of injury to predict PPCS status ≥ 21 days after injury; and (2) assess the ability of salivary ncRNAs measured ≥ 21 days after injury to identify symptom recovery. We investigated longitudinal ncRNA levels and medical/demographic factors among 112 individuals with mTBI at a minimum of two time-points following injury, including an initial saliva sample collected no later than 14 days post-injury and a follow-up sample used to define presence/absence of PPCS, beginning ≥ 21 days post-injury. We hypothesized that an algorithm employing saliva ncRNA levels alongside medical/demographic factors would predict PPCS and identify symptom recovery. Refinement and validation of the algorithm could promote objective anticipatory guidance, facilitate safer return-to-play decisions, and foster effective therapeutics based on individual biologic responses to mTBI.

## Methods

### Ethics

Ethical approval for this study was provided through a central institutional review board (Western IRB 1271583). Written, informed consent was obtained for all participants. Written assent was provided by participants under 18 years of age. The study was registered in the clinicaltrials.gov registry (NCT02901821).

### Participants

This multicenter study included 112 individuals, ages 8–24 years, with a clinical diagnosis of mTBI, as defined by the 2016 Concussion in Sport Group [15]. The participants were enrolled from emergency departments, sports medicine clinics, urgent care centers, concussion speciality clinics, and outpatient primary care clinics at initial clinical presentation (within 14 days of injury) and were repeatedly assessed for symptoms, balance, cognitive test performance, and saliva ncRNA levels up to 60 days post-injury. The cohort was divided into PPCS (*n* = 32) and non-PPCS (*n* = 80) groups based on self-reported symptom scores. PPCS was defined using the upper 95% confidence interval of the mean symptom severity score on the Post-Concussion Symptom Scale (PCSS) from 170 age-matched participants without mTBI (score ≥ 5) [16]. The first symptom report ≥ 21 days post-injury was used to determine PPCS status. A cut-off of 21 days was chosen based on the literature showing that the majority of children (75.6%) report concussion recovery within two weeks, but symptom change flattens between two and four weeks [17]. This threshold resulted in a percentage of PPCS participants (28.6%; *n* = 32) consistent with existing literature [18, 19]. Participants were enrolled at six institutions: Adena Health System (*n* = 14), Colgate University (*n *= 7), Penn State College of Medicine (*n* = 69), State University of New York (SUNY) Buffalo Medical University (*n* = 3), SUNY Upstate Medical University (*n* = 3), and Vanderbilt University (*n* = 16). Participants meeting the following criteria were excluded: non-English speaking, neurologic injury (e.g., intracranial bleeding, spinal cord injury, skull fracture), periodontal disease, upper respiratory infection, secondary oropharynx injury, baseline hearing/vision loss, and drug or alcohol dependency. Additional exclusion criteria included presentation for clinical care > 14 days after injury (*n* = 17), incomplete symptom reports necessary for PPCS classification (*n* = 111), and falling outside the desired age range (*n* = 16; Supplemental Fig. 1).

Samples were divided into a training set (184 samples (58% of total); PPCS = 53, non-PPCS = 131), an evaluation set (72 samples (23% of total); PPCS = 27, non-PPCS = 45), and a semi-naïve testing set (62 samples (19% of total); PPCS = 18, non-PPCS = 44). The training set was used for ncRNA feature selection and algorithm creation. The testing set was used to validate the accuracy of resulting predictive algorithms. The evaluation set was used to minimize bias that could arise from class imbalance by shifting the probability threshold of the classifier away from the standard value of 0.5, while avoiding artificial performance inflation [20]. While the samples in the testing set were naïve, a subset of the participants from which they derive were not (i.e., 37/112 participants were represented in both training and testing sets). Samples were grouped by age, sex, and PPCS-status and assigned randomly across sets. A maximum of five samples per participant were allowed in training and testing sets, with remaining samples being incorporated into the evaluation set. First, the prognostic accuracy of ncRNAs was compared against the Zemek 12-point risk score [21], employing samples with complete data for “history of concussion” and “medical diagnosis of chronic headaches or migraines” in addition to age, sex, and select symptom information (218 samples, PPCS = 62, non-PPCS = 156). Next, the ability of ncRNAs to differentiate recovered and non-recovered participants ≥ 21 days post-injury was compared against computerized cognitive test and balance scores (77 samples, PPCS = 17, non-PPCS = 60).

### Measures

Medical/demographic characteristics were collected from each participant via survey at enrollment. For children ≤ 12 years of age, parents assisted with survey completion. Concussion-related symptoms were self-reported on a 7-point scale (0–6) using the PCSS [22]. These survey characteristics enabled recapitulation of all nine predictors (each having 0–2 risk points for PPCS) from the Zemek 12-point risk score model. The nine predictors and PCSS counterparts are: age group (three bins, 5–18), sex, prior concussion and symptom duration, migraine history, answering questions slowly (“Feeling slowed down”), tandem stance balance errors (“Balance problems”), headache (“Headache”), sensitivity to noise (“Sensitivity to noise”), and fatigue (“Fatigue or low energy”). Balance and cognitive function were assessed using the ClearEdge system (Quadrant Biosciences Inc., Syracuse NY) [23]. Body sway was measured in eight stances: two-legs eyes-open (TLEO), tandem-stance eyes-open (TSEO), two-legs eyes-closed (TLEC), tandem-stance eyes-closed (TSEC), two-legs eyes-open on foam pad (TLEOFP), two-legs eyes-closed on foam pad (TLECFP), tandem-stance eyes-open on foam pad (TSEOFP), and tandem-stance eyes-closed on foam pad (TSECFP). The computerized cognitive assessment included simple reaction time (SRT1), procedural reaction time (PRT), go/no-go (GNG), and a repeat of simple reaction time (SRT2) [24]. The Minimal Detectable Change (MDC) value [25, 26] for cognitive and balance tests were used to determine whether a participant’s change in performance from enrollment to follow-up was a real change, or whether it fell within the 95% confidence interval of random measurement error. As we have previously described [16], non-fasting saliva samples (*n* = 505) were collected from all participants (*n* = 112) using OraCollect Swabs (DNA Genotek, Ottowa Canada). RNA sequencing was performed at a depth of 10 million reads per sample, using 50 base-pair single end reads, on an Illumina NextSeq 500 instrument. Fastq files were aligned to the following databases: miRBase22 (miRNAs), RefSeq v90 (small nucleolar RNAs; snoRNA), and piRBase v2 (piwi-interacting RNA). To allow for efficient and meaningful alignment from piRBase, highly similar sequences were reduced using hierarchical clustering. Resulting sequences were termed wiRNAs. Aligned reads were filtered to remove low counts (< 0.01% of total reads per RNA category), normalized using total sum scaling, and inverse hyperbolic sine transformed to correct for skew.

### PPCS versus non-PPCS comparisons

Three ncRNA sub-types (miRNA, snoRNA, wiRNA) were compared among PPCS and non-PPCS groups at two time-points: (1) initial (≤ 14 days post-injury) and (2) follow-up (≥ 21 days post-injury). Mean symptom, balance performance, and cognitive performance scores were also compared between PPCS and non-PPCS groups at each time-point. To identify changes in ncRNA levels during “typical” recovery, a paired test looked at differentially expressed RNAs within the non-PPCS group only, comparing their “follow-up” and “initial” samples.

### Feature selection

Training data were processed through a custom, multifold feature selection pipeline in R (*caret* package) consisting of neural network- and random forest-based algorithms. Top features appearing in > 50% of the folds were combined with ncRNAs identified from differential expression and penalized generalized linear model (GLM) analyses. Penalized GLMs identified ncRNA predictors associated with symptom scores, balance and cognitive test performance, and injury-associated risk factors. RNAs with significant Pearson correlation coefficients (*p* < 0.05, unadjusted) were chosen from linear regression models (for numeric response variables), along with the three highest ranked RNAs in terms of variable importance from logistic regression models (for binary response variables) with “fair” predictive accuracy (kappa > 0.20). The reduced feature set was used to train the PPCS algorithm (see below). Recursive feature elimination was used to further refine the panel. At each iteration, the feature resulting in maximum weighted algorithm performance upon omission was removed until optimal performance was reached. A gradient-boosted machine (GBM) model was used to rank the final features in order of importance.

### Prognostic algorithm development

To create a prognostic algorithm capable of predicting PPCS status, a training set of 113 non-PPCS and 53 PPCS samples (collected within 14 days of injury from 72 and 28 participants, respectively) was used to train a radial support vector machine (rSVM) algorithm (Supplemental Table 1a). Performance was evaluated using AUC from repeated tenfold cross-validation along with sensitivity, specificity, positive predictive value, and negative predictive value. A naïve testing set of 44 non-PPCS and 18 PPCS samples from 33 and 16 participants, respectively (Supplemental Table 1b), was used to validate algorithm performance. Stratified random sampling in R was used to ensure age- and sex-matching of the PPCS and non-PPCS groups across the training, evaluation, and testing sets, as well as equal % PPCS across sets. Sampling was performed only once to avoid bias and to maintain a truly naïve testing set. To compare the ncRNA algorithm with an existing clinical assessment tool, rSVM models were trained using features from the Zemek 12-point risk score model. Performance was assessed through AUC on tenfold cross-validation. A third model was generated combining the risk score with ncRNAs.

### Identifying mTBI recovery

The same feature selection pipeline was used to select ncRNAs capable of objectively identifying individuals with symptom recovery. In addition to age, individual cognitive and balance test scores were used as features in a random-forest model. Predictive capability of cognitive and balance testing was compared with that of ncRNAs by performing repeated tenfold cross-validation. The cross-validation approach was chosen due to the reduced number of participants (78/112) for whom complete balance and cognitive test results were available at initial and follow-up time-points. To increase fidelity of group assignment, samples with an associated PCSS score within two of the threshold score (*n* = 5) were also excluded. A set of 60 non-PPCS and 17 PPCS samples from 58 and 15 participants, respectively, was used. (Supplemental Table 1c).

### Statistical analysis

R version 3.6.1 was used for all statistical analyses. The data were analyzed by paired (e.g., initial vs. follow-up time points) or unpaired (e.g., PPCS vs. non-PPCS) t tests, one-way ANOVA in the case of multiple groups, or the Mann–Whitney test in case of nonparametric distribution. A Chi-squared test with Yates correction was used for nominal data. Differential expression analysis was performed using the DESeq2 package (version 1.24.0), where *p* values were attained by the Wald test. Multiple testing correction was achieved with the Benjamini–Hochberg method. Algorithm performance was evaluated by AUC and statistically compared using the method of DeLong. Unless otherwise noted, * denotes *p* ≤ 0.05, ** denotes *p* ≤ 0.01, and *** denotes *p* ≤ 0.001.

Power analysis and sample size software (NCSS PASS 2019, Chapter 260) was used to determine that the sample size in the training set provided 99% power to detect a difference between the null AUC = 0.68, taken from the Zemek 12-point-risk score model validation AUC, and the alternative hypothesis, AUC = 0.856, estimated from our previously published research [14]. A two-sided *z* test was used with *α* = 0.05 for continuous data with equal variances and binomial outcomes. The testing cohort achieved 74% power to differentiate the ncRNA model performance (AUC = 0.87) from the Zemek risk score model (AUC = 0.68).

## Results

### Participants characteristics and symptoms

**Fig. 1:**
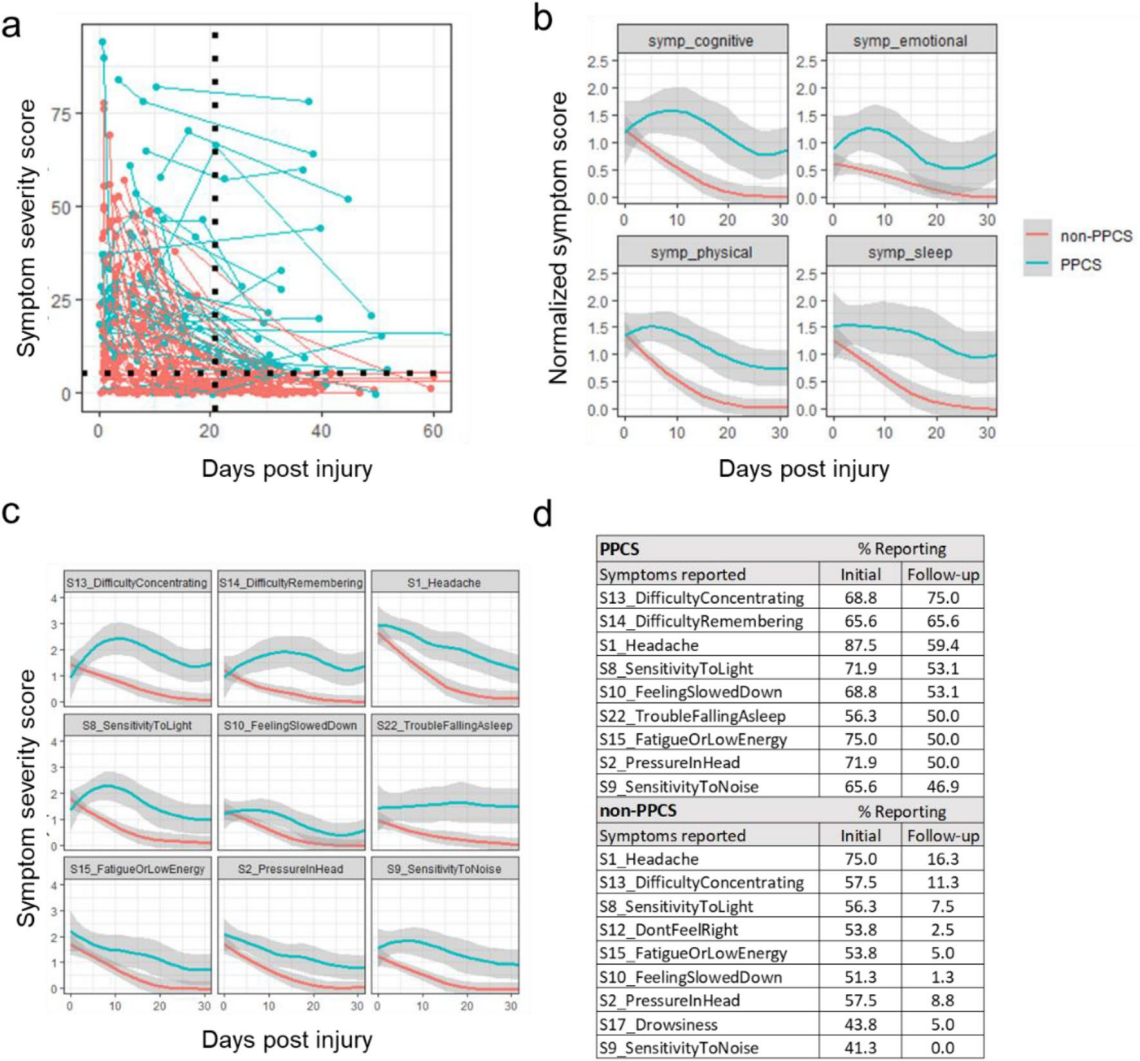
Longitudinal patterns in self-reported symptoms among individuals with or without persistent post-concussion symptoms (PPCS). **a** A scatter plot of symptom severity score versus time post-injury for all study participants. Participants having symptom scores > 5 persisting ≥ 21 days post-injury (black dotted lines) were considered to have PPCS. **b** The 22 PCSS symptoms were grouped and normalized to account for unequal numbers of symptoms per group. Longitudinal symptom scores, normalized by symptom category for PPCS and non-PPCS cohorts, were fit with a local regression and visualized with the 95% confidence intervals (gray). **c** Longitudinal trends for the nine symptoms most commonly reported by the PPCS cohort, grouped by PPCS status. **d** Table comparing the most frequently reported symptoms at initial and follow-up time points for PPCS and non-PPCS participants

Participants had a mean age of 16 (± 4) years (Table 1). Participants included 49 females (44%). Demographic, medical, and concussion characteristics were largely consistent across PPCS (*n* = 32) and non-PPCS (*n* = 80) groups. However, PPCS participants had a higher incidence of chronic headache (*p* = 0.007) and non-PPCS participants had a higher rate of sports-related concussions (*p* = 0.02). Twenty-two participants reported loss of consciousness at the time of injury and 36 reported initial post-traumatic amnesia. One-third of the participants reported having previous concussions, with the majority of those (59%) having only a single prior concussion. There were 32 participants with PPCS (symptom scores > 5 persisting ≥ 21 days post-injury; Fig. 1a). The PPCS group displayed more gradual symptom resolution (i.e. slower recovery), whether the 22 symptoms were divided into categories (Fig. 1b)—cognitive, emotional, physical, and sleep [27]—or analyzed individually (Fig. 1c). Headache was the most common initial symptom in both PPCS and non-PPCS groups, reported by 88% and 75% of participants, respectively (Fig. 1d). The most common symptom persisting ≥ 21 days post-injury for PPCS participants was “difficulty concentrating” (75%).

**Table 1:** Participant characteristics

	Total (*n* = 112)	non-PPCS (*n* = 80)	PPCS (*n* = 32)	*p* value
Total participants	112	80	32	
Total samples	505	351	154	
Demographic				
Female (%)	49 (44)	34 (43)	15 (47)	0.83
Age, mean (SD)	16.1 (3.7)	16.5 (3.5)	15 (4)	0.06
White (%)	52 (87)	37 (84)	15 (94)	0.58
BMI (SD)	24.2 (6.0)	24.5 (5.6)	23.4 (7)	0.41
Medical				
ADHD (%)	4 (4)	4 (5)	0 (0)	0.48
Anxiety (%)	1 (1)	1 (1)	0 (0)	1.00
Depression (%)	2 (2)	1 (1)	1 (3)	1.00
Chronic headaches (%)	10 (9)	3 (4)	7 (23)	0.01
Concussion characteristics				
Days since injury, initial assessment (SD)	5 (3.6)	4.9 (3.4)	5.2 (4.1)	0.77
Sports cause (%)	82 (73)	64 (80)	18 (56)	0.02
Football cause (%)	34 (30)	27 (33)	7 (22)	0.31
Loss of consciousness (%)	22 (20)	12 (16)	10 (31)	0.12
Post-traumatic amnesia (%)	36 (65)	20 (56)	16 (80)	0.17
Previous concussion (%)	36 (33)	25 (32)	11 (36)	0.70
Number of previous concussions (SD)	1.5 (0.7)	1.4 (0.6)	1.8 (0.8)	0.23
1 previous concussion	16 (59.3)	12 (66.7)	4 (44.4)	
2 previous concussions	8 (29.6)	5 (27.8)	3 (33.3)	
3 previous concussions	3 (11.1)	1 (5.6)	2 (22.2)	
Source				
Penn State College of Medicine—Hershey	69	48	21	
Adena Bone and Joint Center	14	10	4	
Colgate University	7	5	2	
SUNY University at Upstate	3	0	3	
Vanderbilt University	16	14	2	
SUNY University at Buffalo	3	3	0	

Note that immediate post-concussion symptom reports (i.e., loss of consciousness, amnesia) were available for only 56 participants. Medical characteristics were collected via parent/child report, and validated via electronic medical records where available

### PPCS vs. non-PPCS: balance, cognition, saliva ncRNA

**Fig. 2:**
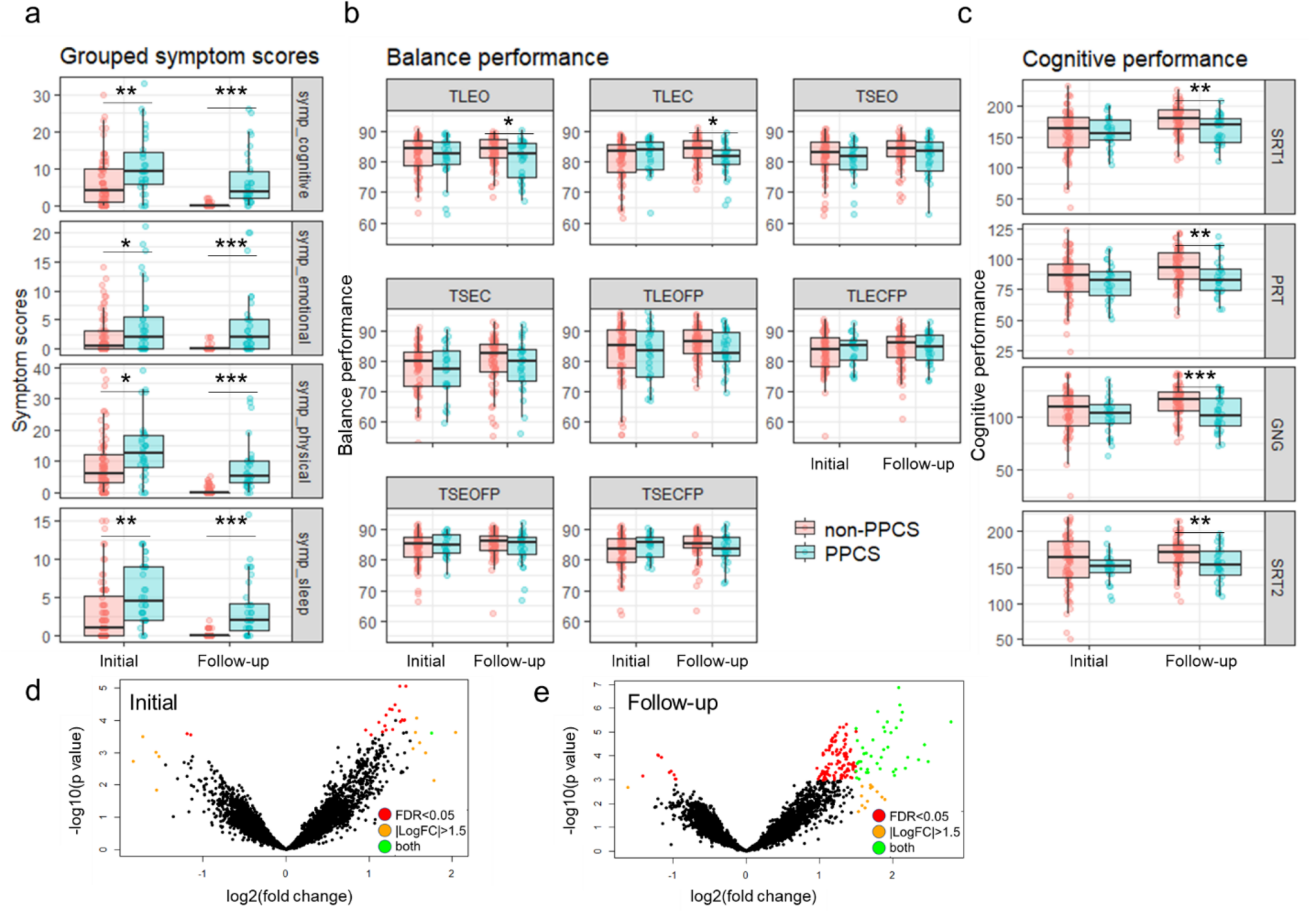
Differences in balance, cognition, and salivary RNA levels between PPCS and non-PPCS participants emerge ≥ 21 days post-injury. **a** Box and whisker plots comparing grouped symptom scores between PPCS and non-PPCS participants at both initial (< 14 days) and follow-up (≥ 21 days post-injury) time points. **b** Plot comparing balance test performance between PPCS and non-PPCS groups across eight different tests at initial and follow-up time points. **c** Plot comparing cognitive test performance between PPCS and non-PPCS groups across four different tests. **d**, **e** Volcano plots comparing RNA abundance between PPCS and non-PPCS subjects at initial and follow-up timepoints. Statistical significance, − log10(*p* value), was plotted against the log2(fold change). A false discovery rate of 0.05 (red) and absolute fold change > 1.5 (yellow) were used as significance cut-offs. ncRNAs passing both criteria are shown in green. *p ≤ 0.05, **p ≤ 0.01, and ***p ≤ 0.001

Initial symptom burden was higher in the PPCS group for each of the four symptom categories (Fig. 2a). At the initial visit, none of the balance or neurocognitive test scores revealed significant differences between PPCS and non-PPCS groups. However, ≥ 21 days post-injury, two of the balance tests (TLEO, TLEC) and all four cognitive tests (SRT1, PRT, GNG, SRT2) differed (Fig. 2b, c). Most PPCS participants did not display improvement between the two time points, while most non-PPCS participants significantly improved on at least two tests (Supplemental Fig. 2A). The difference in improvement between groups was most evident in three cognitive scores: PRT, SRT1, SRT2 (Supplemental Fig. 2B). Supplemental Fig. 3 displays the progression in cognitive, balance, and symptom improvement that occurred for non-PPCS participants (A–C). Notably, PPCS participants exhibited some improvement in all subjective symptom categories, except emotional symptoms (Supplemental Fig. 4A). They did not display improvements in cognition or balance (Fig. 4b, c). Differential expression analysis revealed ncRNA differences between PPCS and non-PPCS groups that became more distinguishable over time, mirroring changes in balance and cognition (Fig. 2d, e). Most between-group differences involved increased levels of piRNA clusters (wiRNAs) among PPCS participants.

### Predictive modeling

**Fig. 3:**
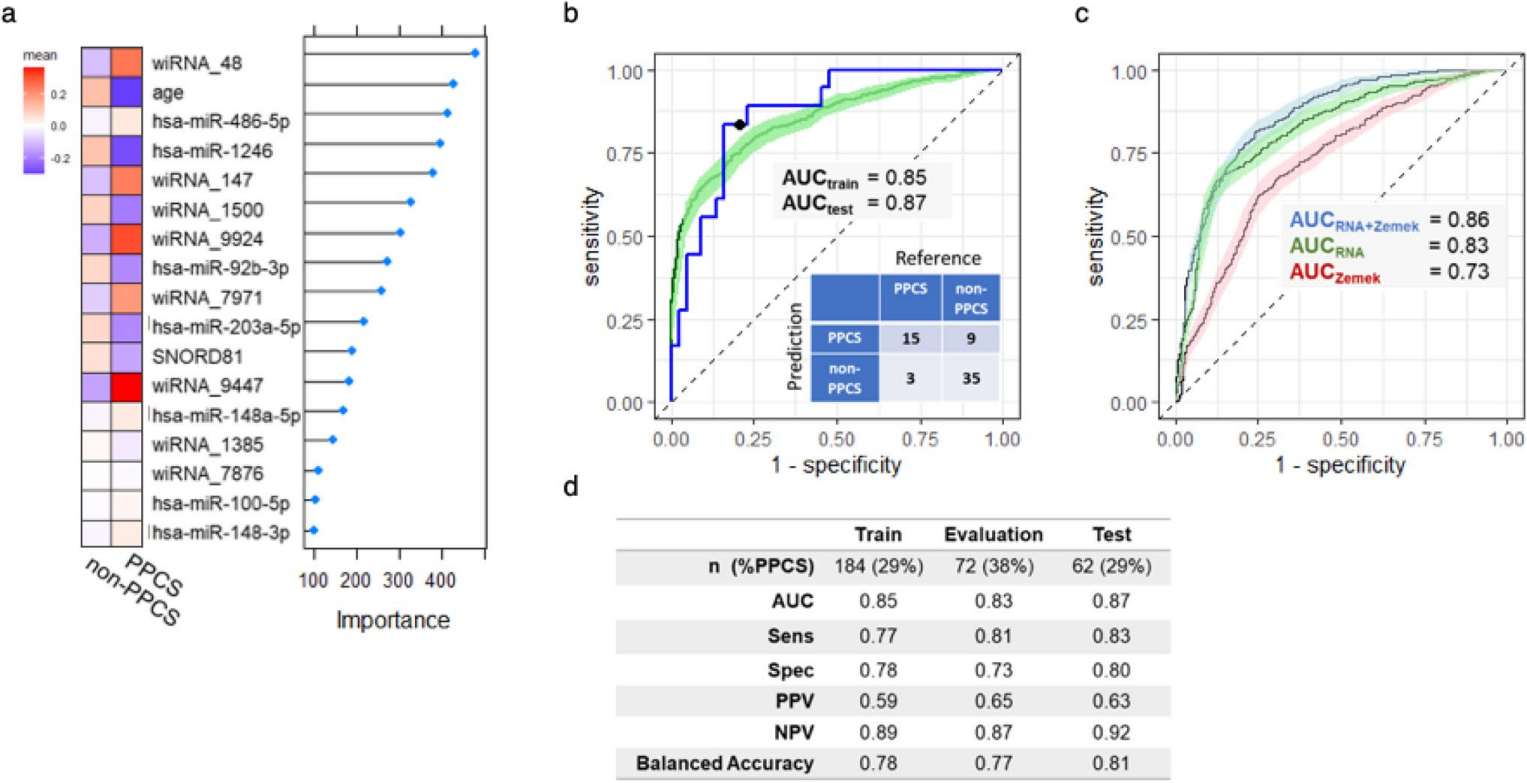
Predicting PPCS risk. A model employing 16 small non-coding RNAs and age accurately predicted PPCS **a**. A GBM algorithm was used to rank model features in order of variable importance. Normalized counts were scaled across RNAs, averaged across PPCS class, and plotted as a heat map to illustrate relative abundance. **b** A receiving-operating characteristic (ROC) curve demonstrates the ability of a rSVM classifier to identify PPCS in a training (green) and testing (blue) set. The testing confusion matrix and AUCs are reported in the plot. **c** ROC curves comparing the performance (AUC) of the RNA PPCS model (“RNA”) with a clinical standard (“Zemek”), as well as an additive model (“RNA + Zemek”). Performance was evaluated using tenfold cross-validation repeated 10 times. The 95% confidence intervals were calculated using the method of DeLong. **d** Table showing the sample breakdown and performance characteristics for the training, evaluation, and testing sets. Sensitivity, specificity, positive (PPV) and negative (NPV) predictive values, and balanced accuracy were calculated using a probability threshold of 0.26, which was optimized using the evaluation set

A combination of machine learning techniques was used to identify ncRNA features whose levels best predicted PPCS status when measured within 14 days of concussion. The final algorithm included 16 ncRNA features (seven miRNAs, one snoRNA, eight piRNA clusters) and age (Fig. 3a), and achieved a testing AUC of 0.87 (Fig. 3b, c). Post-hoc analysis revealed that individuals contributing multiple swabs (*n* = 37) did not display improved rates of prognostic accuracy, with 79% classification accuracy versus 86% for samples from naïve participants (Supplemental Fig. 6B). To understand how the ncRNA classifier compared with a conventional clinical tool for assessing PPCS risk, we optimized a rSVM model using nine features from the 12-point clinical risk score for PPCS [21]. Figure 3d shows the results of a tenfold cross-validation, comparing the clinical risk score with the ncRNA model. The performance of the ncRNA model (AUC = 0.83; 95% CI 0.81–0.85; Supplemental Table 2) was superior to that of the modified clinical risk score (AUC = 0.73; 95% CI 0.70–0.75) by DeLong’s test for two ROC curves (*p* = 1.05e−10). The model combining ncRNAs with clinical risk features performed best (AUC = 0.86; 95% CI 0.84–0.88), surpassing the ncRNA model (*p* = 3.24e−05).

**Fig. 4:**
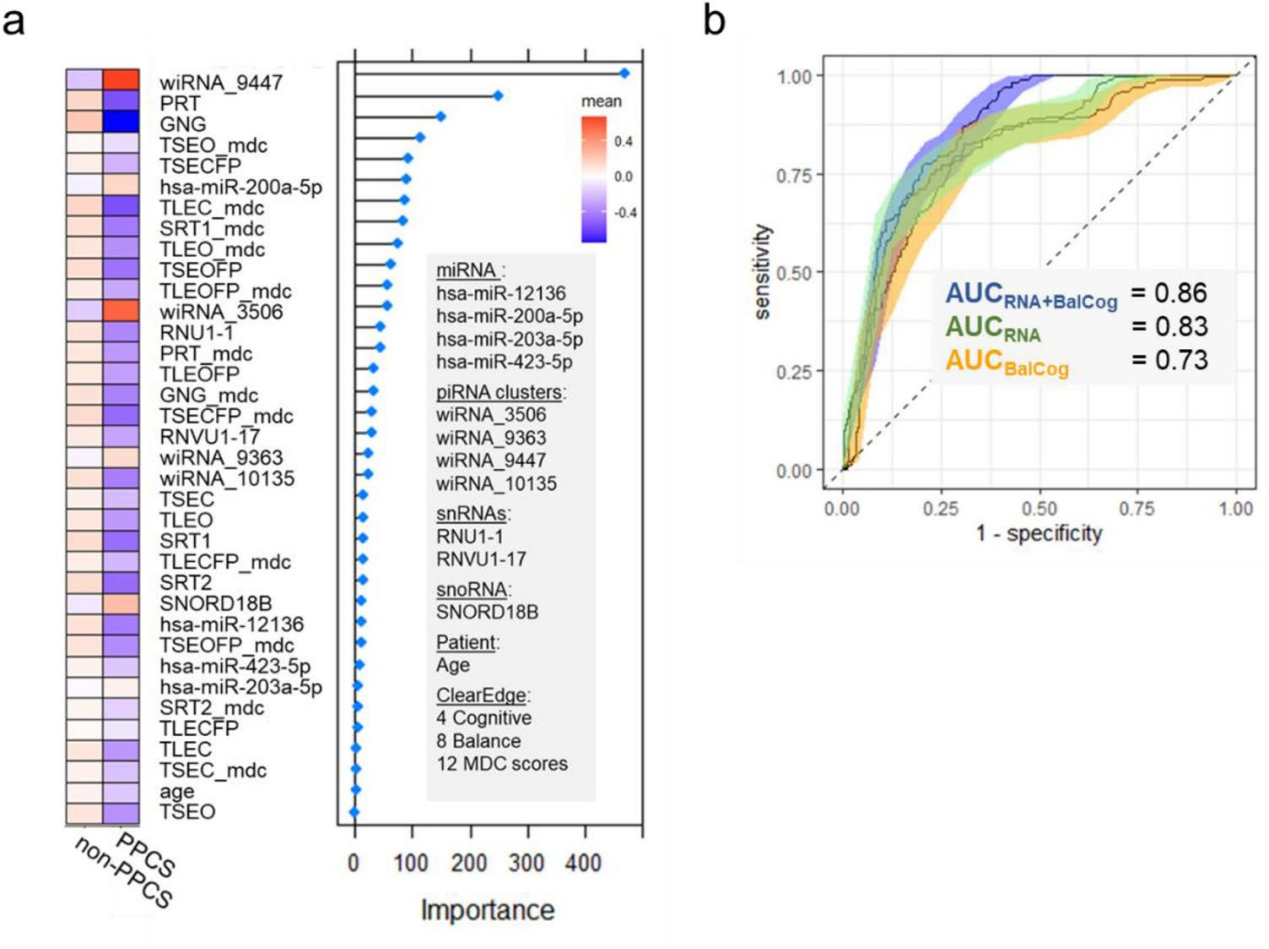
Identifying mTBI recovery using balance, cognitive, and ncRNA measures. **a** 11 RNAs, eight balance test scores, four cognitive test scores, and age were used to determine mTBI recovery with high accuracy (AUC = 0.86). The Clear Edge platform was used for objective measurement of balance and cognition. **b** ROC curve showing the ability of three random forest classifiers to classify recovered participants at ≥ 21 days, using either (1) 12 balance and cognitive test scores and age (“BalCog”), (2) 11 RNA features and age (“RNA”), or (3) an additive model combining 1 and 2 (“RNA + BalCog”). Performance was evaluated using tenfold cross-validation repeated 10 times. The 95% confidence intervals were calculated using the method of DeLong

The ability of ncRNA levels ≥ 21 days post-injury to identify symptom recovery was compared against balance and cognition. The best performing ncRNA model consisted of four miRNAs, four wiRNAs, three snoRNAs and age (Fig. 4a). The heat map of GBM-ranked “recovery” features in Fig. 4a demonstrates that individuals with PPCS have poorer balance and cognitive scores at ≥ 21 days post-injury compared to non-PPCS counterparts. The balance/cognition model displayed an AUC of 0.79 (95% CI 0.76–0.83) for differentiating “recovered” and “non-recovered” participants. The ncRNA model displayed an AUC of 0.83 (95% CI 0.79–0.86). A model combining balance, cognition, and ncRNA levels displayed an AUC of 0.86 (95% CI 0.83–0.89) (Fig. 4b). The combined model performed significantly better than the balance/cognition model (*p* = 0.006), but not the ncRNA model (*p* = 0.16).

### Associations between ncRNAs and clinical features

The relationships of prognostic and recovery ncRNAs with symptom reports and measures of balance and cognition were modest (Supplemental Fig. 5A, B). The only ncRNA associated with a functional test was miR-148a-5p, which was associated with PRT (Table 2). Several ncRNAs were significantly associated with self-reported symptom scores: seven (none from the predictive models) with “sensitivity to noise”; six wiRNAs, including the prognostic feature wiRNA 1500, with “more emotional”; and the prognostic feature, miR-205-5p, with “neck pain.” Numerous ncRNAs were associated with time post-injury, four of which were prognostic ncRNAs that also displayed an effect of time on one-way ANOVA (Supplemental Fig. 6D).

**Table 2:** ncRNAs associated with symptom and functional measures

RNA	Score	*r*	*t*	*P*	adj_sig_thresh
**hsa-miR-205-5p**	**Neck pain**	**0.190**	**3.805**	**1.65E−04**	**1.66E−04**
wiRNA_1383	Sensitivity to noise	− 0.158	− 3.146	1.78E−03	1.83E−03
wiRNA_3304	Sensitivity to noise	0.155	3.092	2.13E−03	2.41E−03
SNORD100	Sensitivity to noise	− 0.152	− 3.030	2.61E−03	2.66E−03
SNORD31	Sensitivity to noise	− 0.155	− 3.077	2.24E−03	2.49E−03
SNORD104	Sensitivity to noise	− 0.153	− 3.050	2.45E−03	2.57E−03
RNA5S17	Sensitivity to noise	0.159	3.173	1.63E−03	1.74E−03
RNA5-8SN3	Sensitivity to noise	− 0.158	− 3.144	1.80E−03	2.16E−03
wiRNA_396	More emotional	− 0.228	− 4.592	5.96E−06	8.31E−05
wiRNA_1073	More emotional	− 0.169	− 3.371	8.25E−04	8.31E−04
**wiRNA_1500**	**More emotional**	**− 0.209**	**− 4.199**	**3.33E−05**	**5.81E−04**
wiRNA_3304	More emotional	0.224	4.515	8.41E−06	3.32E−04
wiRNA_6967	More emotional	− 0.206	− 4.132	4.42E−05	7.48E−04
wiRNA_9246	More emotional	0.220	4.429	1.23E−05	4.98E−04
**hsa-miR-148a-5p**	**PRT**	**0.245**	**4.142**	**4.61E−05**	**8.31E−05**
hsa-miR-1290	Days post injury	− 0.132	− 2.975	3.07E−03	5.15E−03
hsa-miR-30E-5p	Days post injury	− 0.214	− 4.890	1.36E−06	2.49E−04
hsa-miR-101-3p	Days post injury	− 0.163	− 3.698	2.41E−04	2.91E−03
hsa-miR-29c-3p	Days post injury	− 0.155	− 3.504	5.00E−04	3.32E−03
hsa-miR-29b-3p	Days post injury	− 0.173	− 3.922	1.00E−04	2.24E−03
hsa-miR-141-3p	Days post injury	− 0.205	− 4.681	3.69E−06	4.98E−04
hsa-miR-15a-5p	Days post injury	− 0.140	− 3.154	1.71E−03	3.99E−03
hsa-miR-17-5p	Days post injury	− 0.173	− 3.921	1.00E−04	2.33E−03
hsa-miR-20a-5p	Days post injury	− 0.161	− 3.654	2.86E−04	3.07E−03
hsa-miR-19b-3p	Days post injury	− 0.144	− 3.258	1.20E−03	3.82E−03
**hsa-miR-203a-5p**	Days post injury	**− 0.131**	**− 2.961**	**3.21E−03**	**5.32E−03**
hsa-miR-203b-5p	Days post injury	− 0.169	− 3.836	1.41E−04	2.57E−03
hsa-miR-203a-3p	Days post injury	− 0.172	− 3.894	1.12E−04	2.41E−03
hsa-miR-193b-3p	Days post injury	− 0.130	− 2.941	3.43E−03	5.48E−03
hsa-miR-451a	Days post injury	− 0.138	− 3.111	1.97E−03	4.07E−03
**hsa-miR-423-5p**	Days post injury	**0.126**	**2.846**	**4.61E−03**	**5.81E−03**
hsa-miR-21-5p	Days post injury	− 0.136	− 3.080	2.19E−03	4.49E−03
hsa-miR-23a-3p	Days post injury	− 0.248	− 5.724	1.80E−08	1.66E−04
hsa-let-7E-5p	Days post injury	0.135	3.051	2.40E−03	4.82E−03
hsa-miR-10b-5p	Days post injury	− 0.167	− 3.780	1.76E−04	2.82E−03
**hsa-miR-1246**	Days post injury	**− 0.162**	**− 3.669**	**2.69E−04**	**2.99E−03**
hsa-miR-26b-5p	Days post injury	− 0.151	− 3.416	6.87E−04	3.49E−03
hsa-miR-28-3p	Days post injury	0.159	3.592	3.60E−04	3.16E−03
**hsa-miR-148a-5p**	Days post injury	**0.168**	**3.814**	**1.54E−04**	**2.74E−03**
hsa-miR-106b-5p	Days post injury	− 0.124	− 2.804	5.25E−03	5.98E−03
hsa-miR-183-5p	Days post injury	− 0.169	− 3.830	1.44E−04	2.66E−03
hsa-miR-29a-3p	Days post injury	− 0.186	− 4.226	2.83E−05	6.64E−04
hsa-miR-30b-5p	Days post injury	− 0.157	− 3.552	4.19E−04	3.24E−03
hsa-miR-151a-3p	Days post injury	0.128	2.880	4.15E−03	5.65E−03
hsa-miR-151a-5p	Days post injury	0.149	3.368	8.15E−04	3.57E−03
hsa-let-7a-5p	Days post injury	0.136	3.072	2.24E−03	4.73E−03
hsa-let-7d-5p	Days post injury	0.196	4.464	9.96E−06	5.81E−04
hsa-miR-23b-3p	Days post injury	− 0.262	− 6.070	2.52E−09	8.31E−05
hsa-miR-221-3p	Days post injury	− 0.133	− 3.004	2.80E−03	5.07E−03
hsa-miR-222-3p	Days post injury	− 0.151	− 3.426	6.62E−04	3.41E−03
hsa-miR-502-3p	Days post injury	− 0.132	− 2.972	3.10E−03	5.23E−03
hsa-let-7f-2-3p	Days post injury	− 0.133	− 3.011	2.73E−03	4.98E−03
hsa-miR-374c-3p	Days post injury	− 0.176	− 3.987	7.69E−05	8.31E−04
hsa-miR-374a-3p	Days post injury	− 0.134	− 3.013	2.72E−03	4.90E−03
hsa-miR-374a-5p	Days post injury	− 0.207	− 4.740	2.79E−06	3.32E−04
hsa-miR-361-5p	Days post injury	− 0.126	− 2.831	4.83E−03	5.90E−03
wiRNA_1436	Days post injury	0.123	2.768	5.86E−03	6.56E−03
**wiRNA_3506**	Days post injury	**0.124**	**2.794**	**5.41E−03**	**6.23E−03**
wiRNA_3828	Days post injury	− 0.123	− 2.768	5.84E−03	6.40E−03
**wiRNA_7971**	Days post injury	**− 0.148**	**− 3.357**	**8.47E−04**	**3.65E−03**
**wiRNA_9363**	Days post injury	**− 0.129**	**− 2.902**	**3.87E−03**	**5.56E−03**
**wiRNA_9447**	Days post injury	**0.142**	**3.214**	**1.39E−03**	**3.90E−03**
RNY4	Days post injury	0.131	2.958	3.24E−03	5.40E−03
RNA5S17	Days post injury	− 0.173	− 3.933	9.59E−05	2.16E−03
RNA5-8SN4	Days post injury	0.148	3.343	8.90E−04	3.74E−03
RNA5-8SN3	Days post injury	0.137	3.095	2.08E−03	4.40E−03

Pearson correlation statistics

Bold signifies ncRNA features from PPCS or recovery algorithms

## Discussion

This study, involving 112 individuals with mTBI, defined an algorithm using salivary levels of 16 ncRNAs (eight wiRNAs, seven miRNAs, one snoRNA) obtained within 14 days of injury that demonstrated prognostic utility for PPCS. The saliva ncRNA model outperformed a validated clinical prediction tool [21], and displayed additive utility when used in combination with the clinical prediction tool. Computerized cognitive and balance tests differed between participants with PPCS and non-PPCS, but these differences did not emerge until ≥ 21 days after mTBI. These assessments were strong indicators of mTBI recovery. They performed comparably to a set of 11 ncRNAs (four miRNAs, four wiRNAs, three snoRNAs).

### Clinical implications

A saliva ncRNA test could provide an objective, biologic adjunct, aiding PPCS prognosis in individuals with mTBI. Guidelines for mTBI management recommend that clinicians screen for PPCS risk factors using validated prediction rules [1]. Though prediction rules are relatively simple to administer and interpret, they can be difficult to implement in busy clinics, have not been widely validated outside of an emergency department settings [28], and are less than 70% accurate [21]. In the current cohort, drawn from multiple clinical settings, combination of the prediction rule with saliva ncRNA levels identified PPCS risk with 81% accuracy. Validation of this dual approach in a larger cohort could improve prognostic accuracy and provide opportunities for the development of early, targeted interventions. It may also yield mechanistic insights about the underlying biology of PPCS.

Guidelines advise health care professionals to use a combination of symptom scales, cognitive testing, and balance to assess mTBI recovery. The rationale for multiple measures is that no single tool strongly predicts mTBI outcome [29], and subjective symptom reports can be manipulated if an individual seeks to expedite or delay return to activities [30, 31]. Even baseline cognitive testing can be “sand-bagged” by competitive athletes [32, 33] who may exhibit “volitional poor performance motivated by desire to subvert concussion detection and potential removal from play" [34] and guidelines acknowledge that evidence for balance testing is limited to older adolescent athletes [35]. Here, we show the relative ability of computerized cognitive assessment, balance testing, and saliva ncRNA to differentiate symptomatic recovery status ≥ 21 days after mTBI. An algorithm incorporating all 8 balance and 4 cognitive test scores accurately differentiated symptomatic individuals from recovered individuals ≥ 21 days after injury (AUC = 0.79). An algorithm employing 11 ncRNAs identified recovered individuals with slightly higher performance (AUC = 0.83). Combining cognitive and balance testing with ncRNAs yielded the best results (AUC = 0.86).

Many miRNAs identified in our previous studies of mTBI were not included in the current predictive model. This likely resulted from our inclusion of piRNA clusters (wiRNAs) that may provide more granular information about PPCS risk, and may therefore be preferentially selected over miRNAs in our machine learning approach. Additional differences between our current and past studies are likely explained by: (1) severity of brain injury (the current study excludes severe TBI); (2) participant age (the current study involves adult participants); and (3) method of sample collection (the current study involves saliva swabs, rather than expectorant).

### Strengths and limitations

To our knowledge this is the largest study of ncRNA in PPCS and among the first to pair longitudinal ncRNA assessment with functional measures. However, several limitations should be acknowledged. The age (predominantly adolescent), race (mostly white), and low rates of anxiety/depression among participants may limit generalizability, despite the fact that individuals were enrolled from six different institutions. Loss of consciousness was slightly more common among participants with PPCS. Although strict clinical criteria excluded participants with severe TBI, the severity of injury among the PPCS group may have been marginally higher and is reflected in symptom scores at the time of injury. However, the PPCS and non-PPCS groups showed no difference in balance or cognitive performance at the time of injury, and these would be expected to differ with TBI severity [36]. We note that complete balance and cognitive data were absent for 34/112 participants, and this may have resulted in selection bias. To boost the predictive power of our study, we employed multiple swabs from a single participant when training and testing ncRNA predictive models. This approach allowed us to use a semi-naïve hold out model, as opposed to the cross-validation used in most prior molecular biomarker studies of PPCS. Though no participants provided > 1 swab on a single day, we acknowledge this approach may have artificially reduced inter-individual variability and increased predictive accuracy of the ncRNA model. We also acknowledge that the hold-out set was still underpowered (74%), thus impacting interpretability. However, sensitivity analyses show that the algorithm is: (1) consistent across training set cross-validation folds (Supplemental Fig. 7, Supplemental Table 3), (2) stable across a range of probability cutoffs (Supplemental Table 4), and (3) robust to outliers (Supplemental Fig. 8, Supplemental Table 5). Although ncRNA accuracy was compared to a validated prediction rule [21], a modified version of this rule was employed because some of our participants fell outside the published age range, a different measure of balance problems was employed, and we did not have complete symptom duration data from prior concussions. We note that performance of the modified prediction rule in our cohort (AUC = 0.73) is similar to published performance of the validated prediction rule (AUC = 0.68).

## Conclusions

Saliva ncRNAs measured within 14 days of mTBI provide prognostic information about risk for PPCS. Combining this novel measure with an existing clinical prediction rule may increase prognostic accuracy for PPCS. Longitudinal measurement of saliva ncRNAs alongside cognition and balance assessment may also improve ability to objectively identify concussion recovery. Such information could aid informed decisions about safe return to activities. Prospective validation of ncRNA measures in a large, diverse cohort would provide additional evidence necessary for clinical adoption of this technology.

## Supplementary Information

Below is the link to the electronic supplementary material.

Supplementary file1 (DOCX 9685 KB)
