# Supplementary material for: Saliva RNA biomarkers predict concussion duration and detect symptom recovery: a comparison with balance and cognitive testing
Source: J Neurol. 2021 May 24;268(11):4349–61. doi: 10.1007/s00415-021-10566-x (PMC8505318; doi:10.1007/s00415-021-10566-x)
Supplement: Supplementary file 1 — Supplementary file1 (DOCX 9685 KB) [file 415_2021_10566_MOESM1_ESM.docx]

**Saliva RNA biomarkers predict concussion duration and detect symptom recovery: a comparison with balance and cognitive testing**

**Journal of Neurology**

Authors: Gregory Fedorchak PhD, Aakanksha Rangnekar, Cayce Onks DO, MS, ATC, Andrea C. Loeffert DO, Robert P. Olympia MD, Samantha DeVita MA, John Leddy MD, Mohammad N. Haider MD, Aaron Roberts MD, Jessica Rieger, Thomas Uhlig, Chuck Monteith MS, ATC, Frank Middleton, PhD, Scott L. Zuckerman, MD, MPH, Timothy Lee, ATC, Keith Owen Yeates, PhD, Rebekah Mannix, MD, PhD, Steven Hicks MD, PhD

Corresponding author –

Steven Hicks

Penn State College of Medicine, Department of Pediatrics, Hershey PA, 17033.

shicks1@pennstatehealth.psu.edu

| Supplemental Table 1 | | | | | | | | | |
| --- | --- | --- | --- | --- | --- | --- | --- | --- | --- |
|  | | | | | | | | | |
| *a. PPCS prognostic model, participant characteristics* | | | | | | | | | |
|  | **Subjects** |  |  |  |  |  |  |  |  |
|  | Train | | | Eval | | | Test | | |
|  | non-PPCS | PPCS | p value | non-PPCS | PPCS | p value | non-PPCS | PPCS | p value |
| Total subjects | 72 | 28 | - | 14 | 7 | - | 33 | 16 | - |
|  |  |  |  |  |  |  |  |  |  |
| Female (%) | 31 (43) | 14 (50) | 0.54 | 6 (43) | 2 (29) | 0.55 | 15 (46) | 7 (44) | 0.91 |
| Age, mean (SD) | 16.4 (3.53) | 15.3 (3.80) | 0.18 | 12.5 (2.01) | 12.5 (3.38) | 0.99 | 15.9 (3.73) | 14.1 (3.88) | 0.15 |
|  |  |  |  |  |  |  |  |  |  |
|  |  |  |  |  |  |  |  |  |  |
|  | **Samples** |  |  |  |  |  |  |  |  |
|  | Train | | | Eval | | | Test | | |
|  | non-PPCS | PPCS | p value | non-PPCS | PPCS | p value | non-PPCS | PPCS | p value |
| Total samples | 131 | 53 | - | 45 | 27 | - | 44 | 18 | - |
|  |  |  |  |  |  |  |  |  |  |
| Female (%) | 53 (41) | 22 (42) | 0.90 | 17 (38) | 8 (30) | 0.48 | 22 (50) | 8 (44) | 0.70 |
| Age, mean (SD) | 15.5 (3.73) | 14.2 (3.76) | 0.03 | 12.4 (2.10) | 12.4 (2.15) | 0.95 | 15.3 (3.64) | 14.0 (3.96) | 0.24 |

| *b. PPCS prognostic cross-validation model + clinical comparison (Zemek 12 point risk score model), participant characteristics* | | | | |
| --- | --- | --- | --- | --- |
|  | **Subjects** |  |  |  |
|  | Data Set | | |  |
|  | non-PPCS | PPCS | p value |  |
| Total subjects | 79 | 31 | - |  |
|  |  |  |  |  |
| Female (%) | 33 (42) | 15 (48) | 0.68 |  |
| Age, mean (SD) | 16.5 | 15 | 0.06 |  |
|  | **Samples** |  |  |  |
|  | Data Set | | |  |
|  | non-PPCS | PPCS | p value |  |
| Total samples | 156 | 62 | - |  |
|  |  |  |  |  |
| Female (%) | 62 (40) | 28 (45) | 0.56 |  |
| Age, mean (SD) | 15.5 | 14.1 | 0.02 |  |

| *c. Recovery model, participant characteristics* | | | | | |
| --- | --- | --- | --- | --- | --- |
|  | **Subjects** |  |  |  |  |
|  | Data Set | | |  |  |
|  | non-PPCS | PPCS | p value |  |  |
| Total subjects | 58 | 15 |  |  |  |
|  |  |  |  |  |  |
| Female (%) | 26 (45) | 8 (53) | 0.57 |  |  |
| Age, mean (SD) | 17 (3.49) | 15.7 (4.29) | 0.27 |  |  |
|  |  |  |  |  |  |
|  |  |  |  |  |  |
|  | **Samples** |  |  |  |  |
|  | Data Set | | |  |  |
|  | non-PPCS | PPCS | p value |  |  |
| Total samples | 60 | 17 |  |  |  |
|  |  |  |  |  |  |
| Female (%) | 28 (47 | 9 (53) | 0.66 |  |  |
| Age, mean (SD) | 17 (3.46) | 16.1 (4.53) | 0.49 |  |  |
| Days post injury | 32.0 (6.55) | 30.7 (8.51) | 0.56 | |  |

| Supplemental Table 2 | |
| --- | --- |
|  | |
| *PPCS prognostic model, additional performance metrics* | |
| Additional PPCS Algorithm Stats | |
| Accuracy: | 0.81 (95% CI: 0.69 - 0.90) |
| Kappa: | 0.57 |
| Sensitivity: | 0.83 |
| Specificity: | 0.78 |
| PPV: | 0.63 |
| NPV: | 0.92 |
| Prevalence: | 0.29 |
| Detection Rate: | 0.24 |
| Detection Prevalence: | 0.39 |
| Balanced Accuracy: | 0.81 |

| Supplemental Table 3 | | | | | | |
| --- | --- | --- | --- | --- | --- | --- |
|  | | | | | | |
| *Performance metrics from PPCS training set, repeated 10-fold cross-validation* | | | | | | |
| cutoff = 0.50 | |  |  |  |  |  |
|  | AUC | | Sensitivity | | Specificity | |
| Rep | M | SD | M | SD | M | SD |
| Rep01 | 0.878 | 0.078 | 0.603 | 0.230 | 0.954 | 0.065 |
| Rep02 | 0.864 | 0.094 | 0.593 | 0.159 | 0.954 | 0.054 |
| Rep03 | 0.880 | 0.100 | 0.640 | 0.285 | 0.932 | 0.055 |
| Rep04 | 0.857 | 0.126 | 0.563 | 0.245 | 0.932 | 0.075 |
| Rep05 | 0.876 | 0.100 | 0.563 | 0.228 | 0.947 | 0.037 |
| Rep06 | 0.870 | 0.085 | 0.623 | 0.165 | 0.962 | 0.054 |
| Rep07 | 0.839 | 0.073 | 0.547 | 0.196 | 0.939 | 0.079 |
| Rep08 | 0.836 | 0.173 | 0.550 | 0.224 | 0.940 | 0.098 |
| Rep09 | 0.822 | 0.094 | 0.573 | 0.117 | 0.940 | 0.069 |
| Rep10 | 0.876 | 0.070 | 0.573 | 0.230 | 0.962 | 0.054 |
|  |  |  |  |  |  |  |
| cutoff = 0.26 | |  |  |  |  |  |
|  | AUC | | Sensitivity | | Specificity | |
| Rep | M | SD | M | SD | M | SD |
| Rep01 | 0.878 | 0.078 | 0.770 | 0.224 | 0.810 | 0.095 |
| Rep02 | 0.864 | 0.094 | 0.760 | 0.168 | 0.802 | 0.074 |
| Rep03 | 0.880 | 0.100 | 0.770 | 0.243 | 0.787 | 0.092 |
| Rep04 | 0.857 | 0.126 | 0.770 | 0.203 | a | 0.148 |
| Rep05 | 0.876 | 0.100 | 0.777 | 0.206 | 0.779 | 0.163 |
| Rep06 | 0.870 | 0.085 | 0.787 | 0.215 | 0.763 | 0.100 |
| Rep07 | 0.839 | 0.073 | 0.753 | 0.163 | 0.779 | 0.082 |
| Rep08 | 0.836 | 0.173 | 0.780 | 0.193 | 0.779 | 0.132 |
| Rep09 | 0.822 | 0.094 | 0.777 | 0.146 | 0.773 | 0.108 |
| Rep10 | 0.876 | 0.070 | 0.777 | 0.136 | 0.748 | 0.136 |
|  |  |  |  |  |  |  |

| Supplemental Table 4 | | | | | | | | |
| --- | --- | --- | --- | --- | --- | --- | --- | --- |
|  | | | | | | | | |
| *PPCS algorithm test performance across a range of probability thresholds* | | | | | | | | |
|  | Probability Threshold | | | | | | | |
|  | 0.10 | 0.20 | 0.22 | 0.24 | 0.26 | 0.28 | 0.30 | 0.40 |
| Accuracy | 0.65 | 0.71 | 0.76 | 0.79 | 0.81 | 0.81 | 0.81 | 0.79 |
| Kappa | 0.37 | 0.43 | 0.50 | 0.56 | 0.57 | 0.57 | 0.57 | 0.48 |
| Sensitivity | 1.00 | 0.89 | 0.89 | 0.89 | 0.83 | 0.83 | 0.83 | 0.61 |
| Specificity | 0.50 | 0.64 | 0.70 | 0.75 | 0.80 | 0.80 | 0.80 | 0.86 |
| PPV | 0.45 | 0.50 | 0.55 | 0.59 | 0.63 | 0.63 | 0.63 | 0.65 |
| NPV | 1.00 | 0.93 | 0.94 | 0.94 | 0.92 | 0.92 | 0.92 | 0.84 |
| Prevalence | 0.29 | 0.29 | 0.29 | 0.29 | 0.29 | 0.29 | 0.29 | 0.29 |
| Detection Rate | 0.29 | 0.26 | 0.26 | 0.26 | 0.24 | 0.24 | 0.24 | 0.18 |
| Detection Prevalence | 0.65 | 0.52 | 0.47 | 0.44 | 0.39 | 0.39 | 0.39 | 0.27 |
| Balanced Accuracy | 0.75 | 0.76 | 0.80 | 0.82 | 0.81 | 0.81 | 0.81 | 0.74 |

| Supplemental Table 5 | | | | | |
| --- | --- | --- | --- | --- | --- |
|  | | | | | |
| *Summary of PPCS algorithm performance after low count outlier inclusion or PCA outlier removal* | | | | | |
|  |  |  |  |  |  |
|  | PCA outliers (n = 4) excluded | | |  | Low count outliers (n = 2) included |
|  | Train | Eval | Test |  | Test |
| n (%PPCS) | 181 (29%) | 71 (38%) | 62 (29%) |  | 64 (29%) |
| AUC | 0.86 | 0.85 | 0.86 |  | 0.86 |
| Sens | 0.74 | 0.81 | 0.83 |  | 0.84 |
| Spec | 0.8 | 0.75 | 0.73 |  | 0.78 |
| PPV | 0.6 | 0.67 | 0.56 |  | 0.62 |
| NPV | 0.88 | 0.87 | 0.91 |  | 0.92 |
| Balanced Accuracy | 0.77 | 0.78 | 0.78 |  | 0.81 |
|  |  |  |  |  |  |
| Prob thresh | 0.29 | | |  | 0.26 |
|  |  |  |  |  |  |

**Supplemental Fig 1**


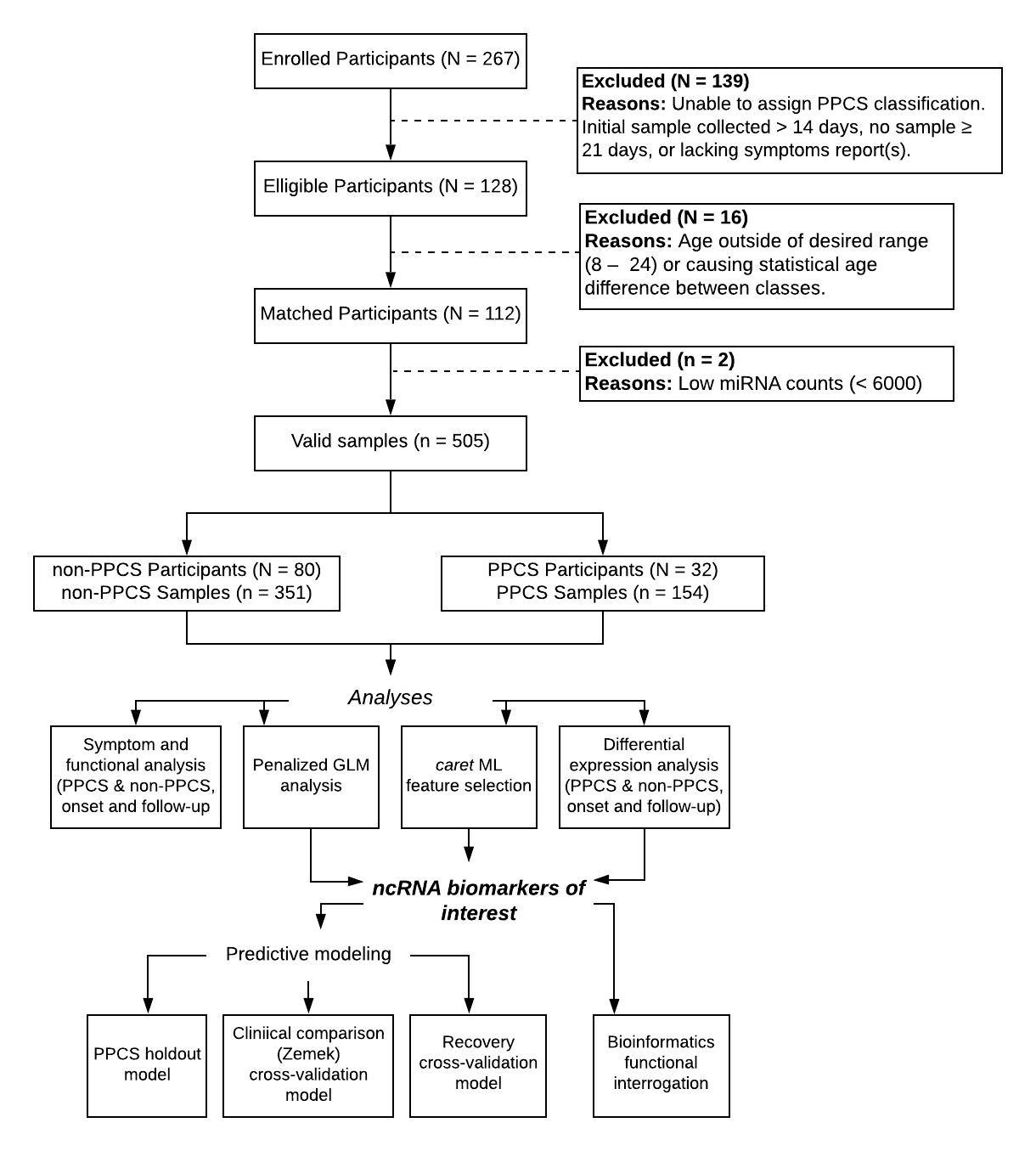


**Supplemental Fig 1 Methodological Pipeline.** Analyses were completed on 505 samples from 112 participants split between PPCS and non-PPCS groups to identify non-coding RNA biomarkers, as well as cognitive and balance features. These features were used to train predictive algorithms to predict PPCS status (at enrollment) and to identify symptomatic recovery (at ≥21 days post-injury).

**Supplemental Fig 2**


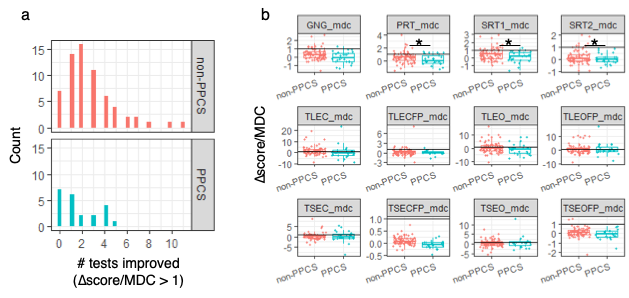


**Supplemental Figure 2 Cognitive and balance changes over time corroborate PPCS classification.** The MDC value for each of the 8 balance and cognitive tests represents the typical intra-participant variance. If the difference between test scores at two time points exceeds the MDC, it is defined as a significant change. **A)** Histograms of the number of tests with significant positive change (e.g., improvement) per person for non-PPCS and PPCS groups. **B)** Scatter plots comparing PPCS and non-PPCS participants that surpass the MDC threshold (horizontal black bar). * denotes p ≤ 0.05.

**Supplemental Fig 3**


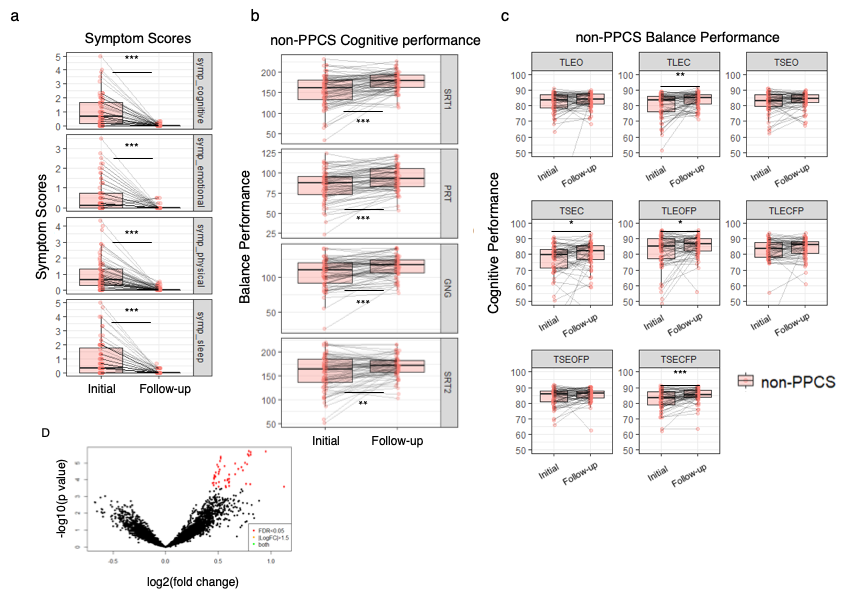


**Supplemental Fig 3 The non-PPCS group exhibits “recovery” in symptoms, balance, neurocognition, and RNA levels.** A paired t-test was used to assess recovery of symptoms, neurocognitive performance, and balance performance among non-PPCS participants (**A-C**). A volcano plot of differentially expressed RNAs among non-PPCS participants at enrollment (symptomatic) and follow-up (recovered) time-points (**D)**. Non-coding RNA levels were generally increased (positive fold change) at follow-up. * denotes p ≤ 0.05, ** p ≤ 0.01, and *** p ≤ 0.001.

**Supplemental Fig 4**


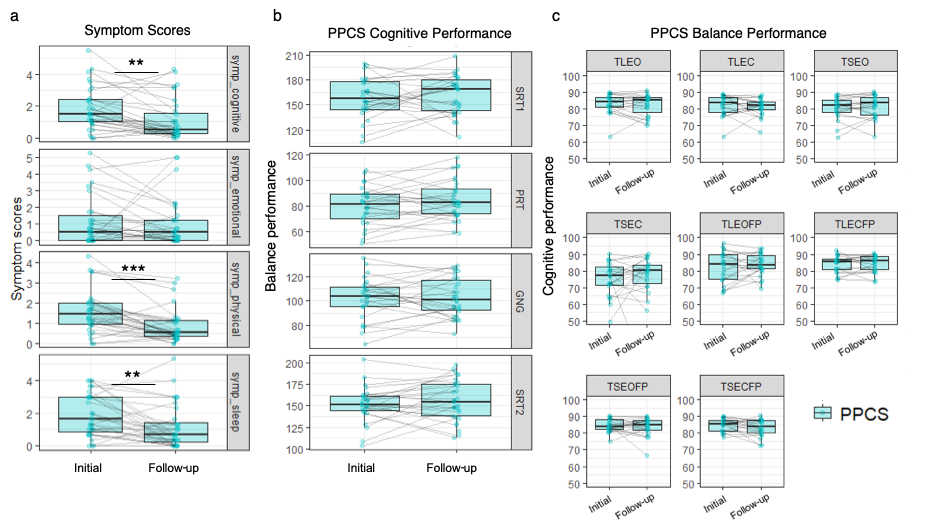


**Supplemental Fig 4 Recovery of balance and neurocognitive function lags subjective symptom recovery in PPCS participants.** A paired sample t-test compared symptom scores, cognitive performance, and balance performance (**A-C)** at initial and follow-up time points. Only participants with scores for both time points for a given test were included. * denotes p ≤ 0.05, ** p ≤ 0.01, and *** p ≤ 0.001.

**Supplemental Fig 5**


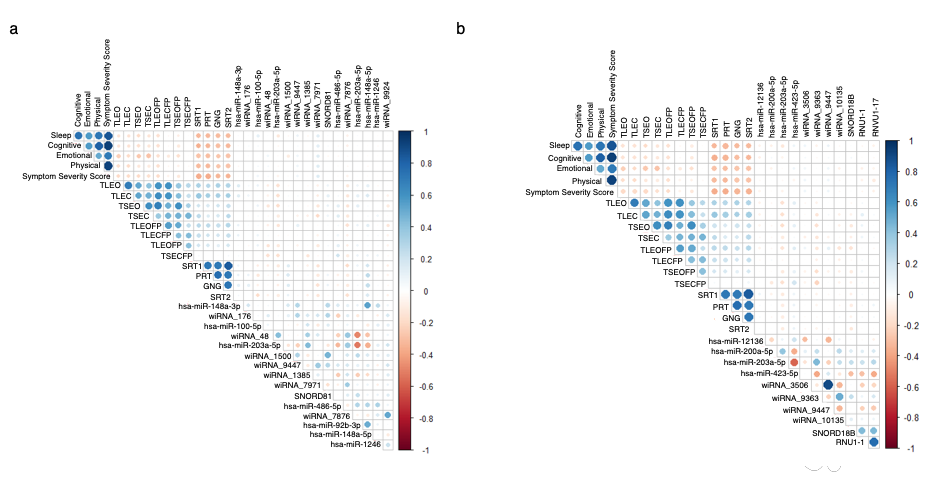


**Supplemental Fig 5 Association of predictive ncRNAs with symptoms, balance, and neurocognitive measures.** Correlation plots showing the Pearson correlation coefficients comparing symptom and functional measures with A) the PPCS prognostic model ncRNA features and B) the recovery model ncRNA features. Positive correlations are displayed in blue and negative correlations in red color. Color and the size of the circle are proportional to the direction and intensity of the correlation coefficients.

**Supplemental Fig 6**


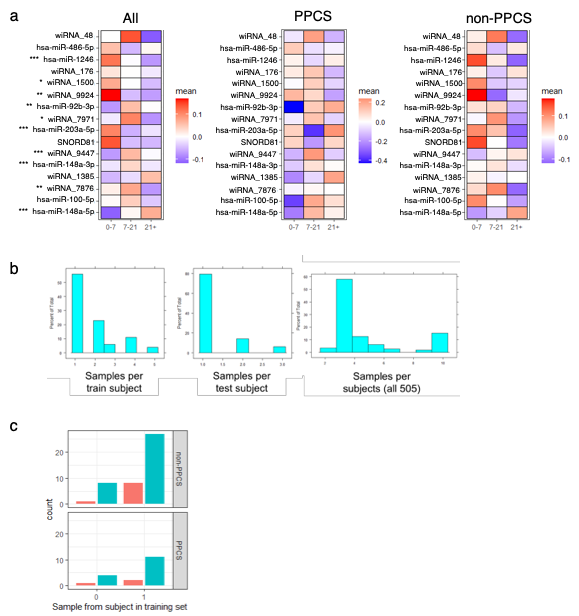


**Supplemental Fig 6 PPCS algorithm supporting information.** A) Heat maps of abundance changes over time for the 16 ncRNA prognostic biomarkers, with asterixis to denote statistical significance by one-way ANOVA. B) Breakdown of samples per participant for the train, test, and overall data set. C) Post-hoc analysis to test whether samples from non-naïve subjects, represented in the training set, are classified more accurately than samples from naïve subjects. The chi-square statistic with Yates correction is 0.026 and the p-value is 0.87.


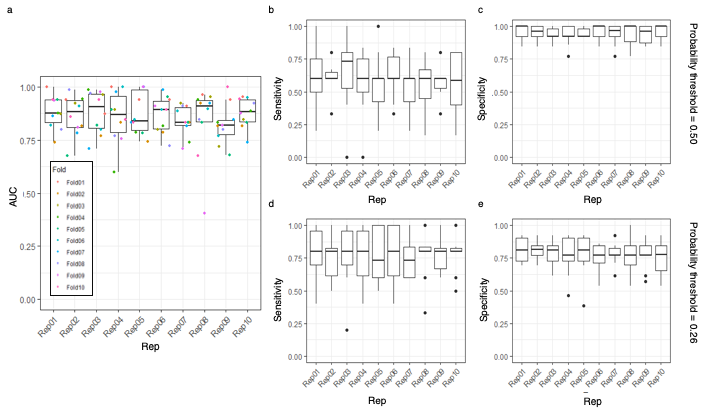


**Supplementary Fig 7 PPCS training set performance from repeated 10-fold cross-validation.** The AUC for each fold and repeat from the training set (n = 184), visualized via boxplot (A). The corresponding sensitivity and specificity distributions were also visualized using boxplots. (B-C) use the default probability threshold (p = 0.50) while (D-E) use the adjusted probability threshold (p = 0.26). Note that AUC is independent of probability threshold. The accompanying data can be found in Supplemental Table 3.


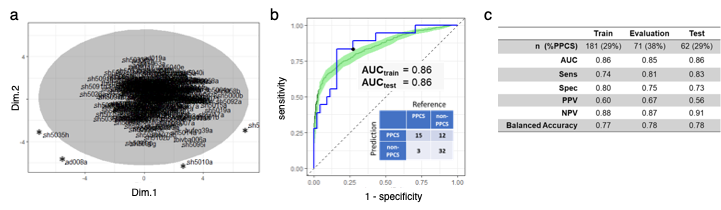


**Supplementary Fig 8. Post-hoc outlier analysis.** A Principle Component Analysis (PCA) was conducted in R (FactoMineR package) using the 17 prognostic algorithm features and all 318 samples from the training, evaluation, and testing sets. A projection onto the first two principal components is shown in (A). Four outlier samples (n = 3 training, n = 1 evaluation) failed to cluster within the confidence ellipse. After outlier omission, algorithm training, evaluation, and testing were repeated and the performance was visualized using a ROC curve with confusion matrix (B) and summarized in table (C). The accompanying data can be found in Supplemental Table 5.
